# Improved Outcomes following the Establishment of a Neurocritical Care Unit in Saudi Arabia

**DOI:** 10.1155/2018/2764907

**Published:** 2018-07-18

**Authors:** Ibrahim Soliman, Waleed Tharwat Aletreby, Fahad Faqihi, Nasir Nasim Mahmood, Omar E. Ramadan, Ahmad Fouad Mady, Babar Kahlon, Abdulrahman Alharthy, Peter Brindley, Dimitrios Karakitsos

**Affiliations:** ^1^Neurocritical Care Unit, Critical Care Department, King Saud Medical City, Riyadh, Saudi Arabia; ^2^Anesthesia and Critical Care Department, Ain Shams University, Cairo, Egypt; ^3^Anesthesia and Critical Care Department, Tanta University, Tanta, Egypt; ^4^Department of Neurosurgery, King Saud Medical City, Riyadh, Saudi Arabia; ^5^Critical Care Department, University of Alberta, Canada; ^6^Department of Critical Care, Keck School of Medicine, USC, Los Angeles, CA, USA

## Abstract

**Background:**

Dedicated neurocritical care units have dramatically improved the management and outcome following brain injury worldwide.

**Aim:**

This is the first study in the Middle East to evaluate the clinical impact of a neurocritical care unit (NCCU) launched within the diverse clinical setting of a polyvalent intensive care unit (ICU).

**Design and Methods:**

A retrospective before and after cohort study comparing the outcomes of neurologically injured patients. Group one met criteria for NCCU admission but were admitted to the general ICU as the NCCU was not yet operational (group 1). Group two were subsequently admitted thereafter to the NCCU once it had opened (group 2). The primary outcome was all-cause ICU and hospital mortality. Secondary outcomes were ICU length of stay (LOS), predictors of ICU and hospital discharge, ICU discharge Glasgow Coma Scale (GCS), frequency of tracheostomies, ICP monitoring, and operative interventions.

**Results:**

Admission to NCCU was a significant predictor of increased hospital discharge with an odds ratio of 2.3 (95% CI: 1.3–4.1; *p*=0.005). Group 2 (*n *= 208 patients) compared to Group 1 (*n *= 364 patients) had a significantly lower ICU LOS (15 versus 21.4 days). Group 2 also had lower ICU and hospital mortality rates (5.3% versus 10.2% and 9.1% versus 19.5%, respectively; all *p* < 0.05). Group 2 patients had higher discharge GCS and underwent fewer tracheostomies but more interventional procedures (all *p* < 0.05).

**Conclusion:**

Admission to NCCU, within a polyvalent Middle Eastern ICU, was associated with significantly decreased mortality and increased hospital discharge.

## 1. Introduction

Neurocritical care (NCC) is an expanding subspecialty within critical care medicine while NCC board certification has been offered since 2007 [1, 2]. NCC units (NCCUs) have become more widespread and have typically evolved from larger multidisciplinary intensive care units (ICUs) into freestanding units. The goal of the NCCU is to optimize care for brain- and spine-injured patients, who can be vulnerable to physiological and biochemical perturbations [3, 4]. Accordingly, a dedicated NCCU—which includes specialized team, protocols, monitoring, imaging, and expertise—may result in less secondary injury and better outcomes [5–7].

There is growing evidence regarding the benefits associated with NCCU-based care for brain-injured patients. These include shorter hospital length of stay and/or better neurological and functional outcomes for all comers [8–12]. Better outcomes have also been reported for specific disease states: cerebral hemorrhage (ICH) [13], acute ischemic strokes [14], subarachnoid hemorrhage (SAH), and traumatic spinal cord and brain injuries [15]. A dedicated NCCU might also be associated with more appropriate resource utilization [14], better adherence to protocols [16], better chart documentation [11], and readmission rates [17]. If so then objective data are important as it could provide justification and leverage for institutions eager to start their own NCCU [18]. Thus far, the vast majority of the NCCU studies have come from North America and Europe, whereas there are scarce data from other nations. This is the first study in the Middle East that evaluated the impact of a newly launched NCCU on the outcome of neurologically injured patients, within the largest polyvalent ICU department in the Middle East.

## 2. Patients and Methods

This study was part of a NCCU performance audit and was approved by the Total Quality Management (TQM) of King Saud Medical City (KSMC). KSMC is the largest ministry of health tertiary referral hospital in Riyadh, Kingdom of Saudi Arabia. The polyvalent KSMC ICU department is the largest in the Middle East (130 operational beds). It is a closed ICU operated 24/7 by consultant intensivists, with an in-house critical care fellow or resident at all times, baring a patient: physician ratio of 12 : 1 and patient: nurse ratio of 1 : 1. In this retrospective cohort study, we compared two time periods: a period of one year prior to the NCCU launching (January 1st to December 31st, 2016) versus a period of nine months after the NCCU was fully operational (January 1st to September 30th, 2017). Patients from the former time period were designated as Group 1, while patients from the latter period were designated Group 2. The latter group included in all neurologically injured patients admitted to the NCCU. In contrast, Group 1 included all neurologically injured patients admitted to the general ICU (since NCCU was not operating at that time) but who fulfilled NCCU admission criteria. These NCCU admission criteria, also served as the study's inclusion criteria, were as follows:

1. Need for intracranial pressure (ICP) monitoring
2. Need for advanced neuromonitoring
3. Need for frequent clinical monitoring due to concerns of neurologic deterioration (including spinal injury)
4. Subarachnoid hemorrhage patients in the vasospasm time window (day 1–14 post-SAH)
5. Complex neurosurgery cases immediately after procedure (as determined by the surgeon)
6. Acute stroke after thrombolytic therapy as well as neuroradiological and/or surgical interventions.

Exclusion criteria applied to both groups were as follows: age ≤18 years old, patients admitted for brain death declaration or in need of solely palliative care, and patients with Do Not Resuscitate (DNR) order. We also excluded patients isolated for infectious conditions (i.e., bacterial meningitis, viral infections, tuberculosis etc.) from Group 1, as the NCCU has no isolation rooms at present, and Group 2 included no such patients. The primary outcome was all-cause mortality and hospital all-cause mortality. Secondary outcomes were ICU length of stay (LOS), identify predictors of ICU and hospital discharge, ICU discharge Glasgow Coma Scale (GCS), as well as the frequency of tracheostomies, ICP monitoring, and operative neurosurgical interventions such as ventriculostomies, craniotomies for hematoma evacuation or removal of contusion, and last tier decompressive craniectomy in TBI and malignant stroke [19, 20]. The study conformed to the principles outlined in the Declaration of Helsinki and was approved by the ICU Ethics Committee.

## 3. Statistical Analysis

Demographic and clinical data were collected retrospectively for all patients from the departmental electronic database and included age, gender, acute physiology, and chronic health evaluation (APACHE) four score and admission diagnosis. Also, we retrieved data of operative interventions in the ICU, ICU LOS, discharge GCS and airway status as well as ICU and hospital outcome. All discrete variables were reported as number (%) and compared with the chi-square test. Continuous variables were reported as mean ± SD and compared with the *t*-test, accounting for unequal sample sizes (Welch's *t*-test) [21]. All tests were two-sided and considered to be statistically significant when *p* value was <0.05.

In a logistic regression analysis to identify predictors of ICU and hospital discharge [22], we used ICU or hospital discharge as a binary outcome measure and admission to NCCU as a predictor adjusted for age, gender, ICU LOS, and APACHE 4 score, whether the patient had experienced trauma or not, and whether an operative intervention occurred or not. The prediction models used enter method with enter *p* value of <0.1 and tested for goodness of fit with Hosmer–Lemeshow test, the calibration of each model was evaluated by the area under the curve (AUC) of receiver operator characteristics (ROC) curve, accepted as good if the AUC ≥ 0.7. All statistical tests were carried out by SPSS^©^ version 21 for Windows (SPSS Inc. Chicago, Illinois, USA).

## 4. Results

In 2016, a total of 2442 patients were admitted to the polyvalent ICU. Of those, 364 patients fulfilled inclusion criteria for NCCU admission. Since no NCCU yet existed, they were admitted in the polyvalent ICU and represented Group 1 in our study. In 2017, 1765 patients were admitted to the ICU in 9 months, with 208 patients admitted to the NCCU and therefore designated as Group 2. The comparative demographics of Groups 1 and 2 are presented in Table 1.

ICU mortality in Group 2 (5.3%) was significantly lower than Group 1 (10.2%) (*p*=0.034), likewise, hospital mortality was significantly lower in Group 2 compared to Group 1 (9.1% versus 19.5%, *p*=0.001), (Table 2). The most common causes of death in Group 1 in the ICU were acute respiratory distress syndrome (ARDS) 43%, followed by brain herniation 30%, and then sepsis and septic shock 27%, whereas Group 2 ICU mortality was mostly due to ARDS 45%, sepsis and septic shock 36%, and brain herniation 19%. Withdrawal of care, however, was statistically similar in both groups although lower in Group 2 (13% in Group 1 and 8% in Group 2, *p*=0.09). Sepsis and septic shock was the most common cause of hospital mortality in both groups (56% in Group 1 and 55% in Group 2). Since the majority of patients in both groups were trauma patients with high severity scores, 62% of Group 1 patients were mechanically ventilated as compared to 61% in Group 2 (*p*=0.9); 61.5% of Group 1 patients required hemodynamic support (>0.05 mcg/kg/min noradrenaline) to maintain the BP targets of perfusion of the acutely injured brain and spinal cord, while 70.2% of Group 2 patients required hemodynamic support (*p*=0.04); the rate of renal failure requiring hemodialysis at least once was not different between groups (31% in Group 1 versus 27% in Group 2, *p*=0.4). Analysis of secondary outcomes revealed decreased ICU LOS in Group 2 compared to Group 1 (*p* < 0.001; Table 2). Group 2 patients exhibited a higher ICU discharge GCS, underwent fewer tracheostomies but had more ICP monitoring and operative neurosurgical interventions compared to Group 1 patients (all *p* < 0.05).

Two multivariate logistic regression models were fitted to evaluate independent predictors of ICU and/or hospital discharge among NCCU patients (evaluated for age, APACHE 4 score, gender, NCCU admission, presence of trauma, LOS, and operative intervention; Table 3). The models revealed that NCCU admission was not significantly correlated to ICU discharge (OR = 1.5; 95% CI: 0.71–3.3; *p*=0.285) but was a significant predictor for hospital discharge with an OR of 2.3 (95% CI: 1.3–4.1; *p*=0.005). Other significant predictors in both models were age and ICU LOS.

**Figure 1:**
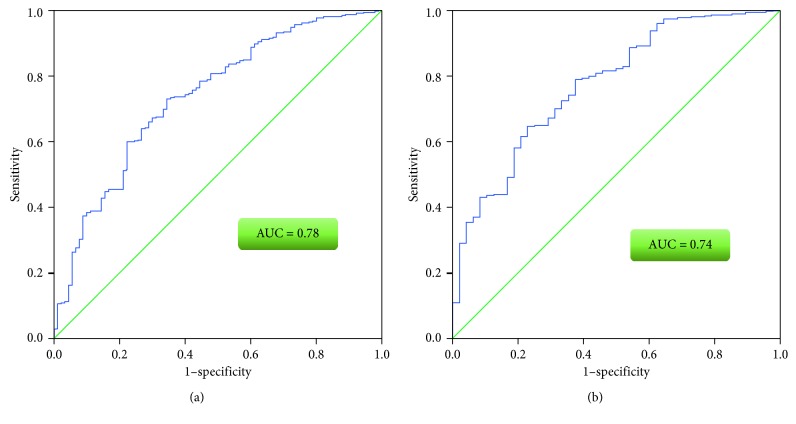
ROC curve of logistic regression models. (a) ICU discharge; (b) hospital discharge.

Both models were well fitted as *p* values of the Hosmer–Lemeshow test were 0.28 and 0.67 in the multivariate logistic regression analysis, respectively. Both models were also well calibrated (evaluated the degree of correspondence between the estimated probabilities of mortality produced by a model and the actual mortality) as evident by the AUC of the logistic regression model for ICU discharge of 0.78 (95% CI: 0.71–0.84) and that of the hospital discharge model of 0.74 (95% CI: 0.68–0.8) (Figure 1).

## 5. Discussion

In this retrospective before and after cohort study, we have shown that establishment of a dedicated NCCU was associated with an increase in meaningful clinical outcomes for neurologically injured patients.

The mortality rate (5.3%) in the NCCU was significantly lower compared to the general ICU (10.2%; *p*=0.034). This mirrors work by Jeong et al. [8]. Other larger studies have reported overall higher rates of NCCU mortality, such as 18% by Broessner et al. [23] (*n *= 1000). Our hospital mortality rate was significantly reduced from 19.5% in general ICU to 9.1% (*p*=0.001) in NCCU. This is also in accordance with findings of Varelas et al. [24], although other studies reported insignificant difference [25]. Adding to the significance of the reduced ICU and hospital mortality rates is the fact that, throughout the study period, there were no changes in our ICU's discharge policy or DNR policy. Furthermore, the general management of critically ill patients was consistent throughout the study period; however, the addition of new standards of management in the form of NCCU specific protocols to maintain better brain and spinal cord perfusion during acute injury guided by more ICP/CPP monitoring (evident by the significantly different rates of hemodynamic support requirement) may explain the difference in outcome objectively as adherence to guidelines was translated to more monitoring which guided us to maintain more perfusion of CNS by using more hemodynamic support in Group 2.

ICU LOS was significantly shorter for patients hospitalized in the NCCU compared to the general ICU (*p* < 0.001), although there were no changes in the setting or discharge policies of our institute nor was a step-down unit or new rehabilitation services established throughout the study period. This also duplicates what others have reported [26, 27]. Notably, we did have a relatively high ICU LOS, which we believe can be largely attributed to the lack of a step-down unit in our institution. Notably, Kurtz et al. [10] reported a longer stay in NCCU, but this may reflect the binary model analysis used in his study, namely, patients were separated in two groups, those admitted less than or more than 10 days.

We found NCCU patients to have a better GCS at discharge. Importantly, many studies [8, 25] evaluate the Glasgow Outcome Scale (GOS) or modified Rankin Scale. Unfortunately, we lacked proper GOS data before the launching of NCCU. We accept this as a study limitation that was remedied by our new NCCU electronic medical records archive. Regardless, GCS at ICU discharge was significantly higher in the NCCU Group 2 compared to Group 1 (*p*=0.025). Also, the rate of tracheostomies was significantly lower in the NCCU Group 2 compared to Group 1 (*p*=0.006). While speculative, this could be partially attributed to the increased GCS, to better overall outcome, or to an evolving strategy towards less tracheostomies. In contrast, Kurtz et al. [10] reported that more NCCU patients (35%) were receiving tracheostomy. However, we are unsure about their airway management strategy and intend to pursue this important question now that we have an established NCCU database. Despite the discrepant tracheostomy rates, our data are otherwise in agreement with Kurtz et al. [10] NCCU patients underwent closer neuromonitoring for secondary brain injuries clinically and through ICP insertions (parenchymal or ventricular) according to unit-specific protocols and guidelines for monitoring of different types of neurologic emergencies; hence, those protocols were applied after intensive educational and training activities for bedside nurses and physicians aiming for prevention and early detection and management of secondary injuries mainly intracranial hypertension [8, 10, 22, 23]. The management included more neurosurgical interventions as craniotomies and decompressive craniectomies for refractory intracranial hypertension cases. However, the aforementioned finding does raise the question of whether more neurointerventions could be attributed to more intense neuromonitoring.

Multivariate models, when adjusted for age, gender, APACHE 4 score, LOS, trauma, and postoperative status, revealed that while NCCU admission was not an independent predictor of ICU discharge (OR = 1.5; 95% CI: 0.71–3.3; *p*=0.285), it was a significant predictor of hospital discharge (OR = 2.3; 95% CI of OR: 1.3–4.1; *p*=0.005). This is in accordance with other studies from other jurisdictions. Diringer and Edwards [13] reported that hospitalization outside of NCCU is associated with increased odds of in-hospital death (OR 3.4; 95% CI: 1.65–7.6). Similarly, Suarez et al. [17] showed that the presence of NCC team is an independent predictor of decreased mortality (OR 0.7; 95% CI: 0.5–1).

### 5.1. Limitations

This study has several limitations including its retrospective single-center design and the inherent weaknesses of any before and after analysis, as well as the aforementioned absence of GOS data or other neurological outcome measures such as modified Rankin Scale (mRS), as well as data of discharge disposition. With that said, we are excited to have shown such a positive impact associated with the establishment of a NCCU within a polyvalent ICU setting. Notably, our multivariate logistic regression analysis was tailored to evaluate general prognostic factors but did not include factors specific for particular neurological conditions such as ICH volume/score, SAH grade, and ischemic stroke type/size.

Finally, the age and gender distribution—although similar in both groups—revealed a preponderance of males with a mean age of about 40 years, that is consistent with previous studies on road traffic accident victims in Saudi Arabia [28] who constitute the majority of our patients, a finding that is although typical of the milieu in Saudi Arabia may affect the generalizability of our findings. Further larger prospective multicenter studies are clearly required to confirm and establish the generalizability of our findings.

## 6. Conclusion

Creation of a dedicated NCCU was associated with a significant reduction in ICU and hospital mortality rates, as well as ICU LOS. Admission to NCCU was an independent predictor of discharge from the hospital. NCCU-discharged brain-injured patients exhibited higher GCS and required more frequently invasive neuromonitoring and other interventional procedures with the notable exemption of performed tracheostomies.

## Figures and Tables

**Table 1:** Study demographics.

Variable	Group 1 (*n *= 364)	Group 2 (*n *= 208)	*p* value
Age (years; mean ± SD)	39.5 ± 18.1	40.3 ± 17.9	0.6

Males (*n* (%))	301 (82.7%)	165 (79.3%)	0.3

APACHE 4 (mean ± SD)	67.9 ± 22.2	70.9 ± 22.5	0.1

Diagnosis:			
*Trauma (n (%))*	257/364 (70.6%)	145/208 (69.7%)	0.9
(i) Polytrauma	129/257 (50.2%)	74/145 (51%)	0.96
With TBI	104/129 (80.6%)	59/74 (79.7%)	0.97
With spinal cord injury	25/129 (19.4%)	15/74 (20.3%)	0.97
(ii)Isolated head injury	128/257 (49.8%)	71/145 (49%)	0.96
Brain contusion	32/128 (25%)	23/71 (32.4%)	0.3
EDH	11/128 (8.6%)	7/71 (9.9%)	0.96
SDH	6/128 (4.7%)	4/71 (5.6%)	0.95
SAH	15/128 (11.7%)	11/71 (15.5%)	0.6
Diffuse brain injury	64/128 (50%)	34/71 (47.9%)	0.9
*Nontraumatic (n (%))*	107/364 (29.4%)	63/208 (30.3%)	0.89
ICH	17/107 (15.9%)	11/63 (17.5%)	0.95
SDH	4/107 (3.7%)	2/63 (3.2%)	0.8
SAH	3/107 (2.8%)	2/63 (3.2%)	0.75
Ischemic stroke	44/107 (41.1%)	19/63 (30.2%)	0.2
Brain tumor	8/107 (7.5%)	7/63 (11.1%)	0.6
Others	31/107 (29%)	22/63 (34.9%)	0.5

NCCU = neurocritical care unit; SD = standard deviation; *n *=  number; APACHE = acute physiology and chronic health evaluation; EDH = extradural hemorrhage; SDH = subdural hemorrhage; SAH = subarachnoid hemorrhage; ICH = intracerebral hemorrhage; ^*∗*^other neurological diagnoses included status epilepticus, encephalopathy, Guillain–Barré syndrome, and transverse myelitis.

**Table 2:** Primary and secondary outcomes.

	Group 1	Group 2	*pvalue*
*Primary outcomes*			
ICU mortality (*n* (%))	37 (10.2%)	11 (5.3%)	0.034
Hospital mortality (*n* (%))	71 (19.5%)	19 (9.1%)	0.001

*Secondary outcomes*			
ICU LOS (days; mean ± SD)	21.4 ± 18.5	15 ± 12.5	<0.001
Discharge GCS (mean ± SD)	11.5 ± 2.6	12.5 ± 2.5	0.025
Tracheostomy (*n* (%))	52 (14.3%)	28 (13.5%)	0.006
ICP monitoring, (*n* (%))	87 (24%)	112 (53.8%)	<0.001
Neurosurgical interventions (*n* (%))	34 (9.3%)	41 (19.7%)	<0.001

NCCU = neurocritical care unit; ICU = intensive care unit; LOS = length of stay; GCS = Glasgow Coma Scale; ICP = intracranial pressure.

**Table 3:** Predictors for ICU/hospital discharge.

	Predictors	Odds ratio	95% CI	*p* value
ICU discharge	NCCU admission	1.5	0.7–3.3	0.3
	Age (years)	0.97	0.96–0.99	0.02
	Gender (male/female)	0.6	0.3–1.2	0.13
	APACHE 4 score	0.98	0.97–1	0.051
	ICU LOS (days)	0.97	0.96–0.98	0.02
	Invasive procedures (%)	1.3	0.4–4	0.6
	Presence of trauma	0.9	0.4–1.8	0.7

Hospital discharge	NCCU admission	2.3	1.3–4.1	0.005
	Age (years)	0.98	0.96–0.99	0.001
	Gender (male/female)	0.7	0.4–1.2	0.2
	APACHE 4 score	1	0.98–1.001	0.064
	ICU LOS (days)	0.98	0.97–0.99	0.001
	Invasive procedures (%)	0.7	0.342–1.44	0.335
	Presence of trauma	1.3	0.8–2.2	0.33

OR = odds ratio; CI = confidence interval; ICU = intensive care unit; NCCU = neurocritical care unit; APACHE = acute physiology and chronic health evaluation; LOS = length of stay.
